# Ethics parallel research: an approach for (early) ethical guidance of biomedical innovation

**DOI:** 10.1186/s12910-020-00524-z

**Published:** 2020-09-01

**Authors:** Karin R. Jongsma, Annelien L. Bredenoord

**Affiliations:** Department of Medical Humanities, University Medical Center Utrecht, Julius Center for Health Sciences and Primary Care, University Medical Center Utrecht, Utrecht University, Utrecht, The Netherlands

**Keywords:** Technology, Medical ethics, Empirical ethics, Organoids, AI, Gene editing, Innovation, Society

## Abstract

**Background:**

Our human societies and certainly also (bio) medicine are more and more permeated with technology. There seems to be an increasing awareness among bioethicists that an effective and comprehensive approach to ethically guide these emerging biomedical innovations into society is needed. Such an approach has not been spelled out yet for bioethics, while there are frequent calls for ethical guidance of biomedical innovation, also by biomedical researchers themselves. New and emerging biotechnologies require anticipation of possible effects and implications, meaning the scope is not evaluative after a technology has been fully developed or about hypothetical technologies, but real-time for a real biotechnology.

**Main text:**

In this paper we aim to substantiate and discuss six ingredients that we increasingly see adopted by ethicists and that together constitute “ethics parallel research”. This approach allows to fulfil two aims: guiding the development process of technologies in biomedicine and providing input for the normative evaluation of such technologies. The six ingredients of ethics parallel research are: (1) disentangling wicked problems, (2) upstream or midstream ethical analysis, (3) ethics from within, (4) inclusion of empirical research, (5) public participation and (6) mapping societal impacts, including hard and soft impacts. We will draw on gene editing, organoid technology and artificial intelligence as examples to illustrate these six ingredients.

**Conclusion:**

Ethics parallel research brings together these ingredients to ethically analyse and proactively or parallel guide technological development. It widens the roles and judgements from the ethicist to a more anticipatory and constructively guiding role. Ethics parallel research is characterised by a constructive, rather than a purely critical perspective, it focusses on developing best-practices rather than outlining worst practice, and draws on insights from social sciences and philosophy of technology.

## Background

Until the 1960’s, ethics as an academic discipline hardly ever dealt with ethical questions of everyday life and had a strong focus on abstract debates such as the nature of morality [1]. Medical practice and the dramatic rise in medical technology gave rise to several pressing ethical questions that increasingly demanded ethical attention and guidance. It led to the birth of bioethics, a field in applied ethics to provide orientation to ethical issues in medical practice [2, 3].

These days, societies and certainly also (bio) medicine become more and more permeated with technology, here used to refer to methods, systems, artefacts and devices which are the result of scientific knowledge that can be used for practical applications [4]. Similarly, the fields of medicine and the biomedical sciences are increasingly influenced by the development and use of new (bio)technologies. Gene editing, organoids, and artificial intelligence (AI) constitute some prominent examples. Such technologies have large potential to increase the possibilities for (bio) medical research, better diagnostics, personalised treatment, more care at home, smarter hospitals, and may potentially cure or prevent the onset of (inheritable) diseases. Simultaneously, emerging biotechnologies are paired with ethical questions that include concerns about effectiveness and safety, but also with regard to the changing understanding of the human body, fairness and equity, identity, governance and societal implications. To deal with ethical questions of technological innovation, a comprehensive approach to pro-actively or parallel guide these technologies into society in an ethically sound way is required.

While scholarly attention has been paid to identifying and anticipating ethical aspects of innovation in other technological fields like engineering and design, [5–7] such an approach has not been spelled out yet for (bio) medical research and innovation. This is remarkable, as there are frequent calls for ethical guidance of biomedical innovation, also by biomedical researchers themselves.(e.g.: [8, 9]) New and emerging biotechnologies require anticipation of possible effects and implications, meaning that the orientation is not evaluative after a technology has already been developed or about scenario’s with hypothetical technologies, but real-time for a real technology. Here, we identify six ingredients that are increasingly adopted by ethicists as tools to study and pro-actively co-produce emerging biotechnologies, but these ingredients are only rarely (if at all) brought together and have not comprehensively been described in the literature. We refer to the combination of these six ingredients as ‘ethics parallel research’, which allows to fulfil two aims: guiding the development process of technologies in biomedicine and providing input for the normative evaluation of such technologies. Parallel refers in this notion to the ethical guidance ‘real-time’ along the development process, and interwoven in the field, rather than a lack of interaction with the field, or only interaction after the technology has been developed. Ethics parallel research can be seen as a pragmatic and constructive approach to scrutinize ethical issues of biomedical innovations parallel or proactively as the field develops, in order to guide the development process of biotechnologies and to provide input for the normative evaluation of such technologies. This approach draws on aspects of bioethics as developed from the 1960s onwards, and is inspired by other disciplines such as Science and Technology Studies (STS), the social sciences and philosophy of technology, to suit the challenges of ethically guiding (bio) medical innovation. In this paper, we outline what we see as the characteristics of ethics parallel research and discuss how we should understand it in the context of (bio-)medical ethics.

## Main text

### Ethics parallel research: 6 ingredients

We identify six ingredients that together can be referred to as ethics parallel research: It concerns (1) disentangling wicked problems, (2) upstream or midstream ethical analysis, it is (3) ethics from within, it includes (4) empirical research, draws on (5) participatory design and it focuses on (6) societal impacts. As we will describe, these aspects are related and even overlap, and taken together, these six ingredients help to provide meaningful guidance in the development of new technologies in health care and convene to identifying the ethical implications as the input for a normative evaluation. We first describe the ingredients of ethics parallel research and we will subsequently reflect on the use of these elements in bioethics.

1. ***Disentangling wicked problems***

Biomedical innovations often invoke fierce public reactions and academic controversies that include a large number of stakeholders. This also means that many different perspectives, questions and implications are involved. The societal and ethical challenges of novel biomedical technologies closely resemble what in STS and in policy studies is known as wicked problems [10]. Wicked problems differ from other problems in the sense that they (a) concern several disciplines and stakeholders on various societal levels and (b) there is no agreement on what constitutes the problem nor on the desired solution. In order to understand what stakes are involved, which concerns the stakeholders have, and which problems are essential, a first step to disentangle wicked problems in the field of (bio) medicine is to unravel the different viewpoints, to identify different stakeholders and to clarify which arguments and interests are involved [11]. The aim is to illuminate and unravel the ‘messy’ debate to separate arguments and values, to recognize whether, and if so which, fallacies have been made, to recognize equivocations and to identify whether there are important questions or positions missing or underrepresented. This can be crucial to break up deadlocked opposition in strongly polarized debates: disentanglement may show that different stakeholders are concerned about different arguments, interpret (epistemic) uncertainties differently or may not be informed by reliable information.

This first step of disentanglement does not presuppose the outcome of such debate yet and aims to identify different stances and arguments, which contributes to making the debate more transparent [12]. Disentangling arguments requires theoretical knowledge to recognize different arguments, for which ethicists are well-equipped: one needs to be able to convene theories and knowledge to new contexts and to different types of arguments. In a second step, the different arguments can be weighed and evaluated based on their coherence, validity and persuasiveness. This second step helps in providing ethical orientation for the debate and the development process of the new technology.

For example, germline gene editing (see Supplemental File 1) spawned ethical and societal debate about its goals and the desirability of its development and application from the very onset. The debate is rich of arguments, stakeholders and positions, with no consensus on how to proceed. There is fundamental disagreement between those who perceive germline modification as a line in the sand, [13] others who argue for regulated policy due to potential collective action problems, [14] while still others view germline modification in line with other reproductive genetic technologies that can offer carriers of hereditary disease the possibility to have healthy children, to those who argue that gene editing research on human embryos is a moral imperative [15]. The problem is wicked, as there is fundamental disagreement about whether the aim of germline genome editing (providing people with healthy, genetically related children) is worthwhile to pursue. In addition, even when one would agree about the desirability of the aim, one can debate whether the approach is appropriate (e.g. whether alternatives are better such as having non-genetically related children by means of gamete donation or having children by means of Preimplantation Genetic Diagnosis). Underlying this discussion are different viewpoints and concerns that depart from completely different understandings of the moral status of the embryo, parental rights and duties, the value of genetic relatedness and attitudes to (reproductive) technology. In addition, in many countries, the application of clinical germline editing would require a law amendment, which makes it not only an academic and public debate but also a political affair. There has also been a call for global governance of germline editing, [8] which means that international collaboration is also required. In other words, apart from mere technological concerns, the alignment between laboratories, clinicians, patients, ethicists, regulators, the general public and international partners will be a bigger challenge for developing germline genome editing. Germline editing is thus an excellent example of a wicked problem, as multiple disciplines and stakeholders are involved, who are needed to determine how (if ever) to proceed responsibly and there are diverging views on both the problem and the solution. This is partly due to the fact that it is an emerging technology, meaning that there is (epistemic) uncertainty about its effects, safety and efficacy, which is inherent to the early stages of development. How such uncertainties are interpreted, however, differ, which underlie the different moral stances of the stakeholders in the debate. In order to assess the ethical acceptability of germline editing, insights into its safety, efficacy, and alternatives are helpful, but also the potential medical indications and moral considerations need to be disentangled. The underlying debate is essentially a question about values, meaning and governance for which clarification about the meanings and use of notions such as human identity, naturalness and equity are a starting point for understanding disagreement and clarifying the debate. Ethicists can bring the debate further by disentangling such wicked challenges and by weighing and evaluating the different moral stances.

(2) ***Upstream or midstream ethical analysis, not merely end of pipeline***

Ethics parallel research focuses on ethical guidance of emerging technologies in order to provide guidance during the development process. The question about the right time of assessment and intervention of a technological development, has been debated since Collingridge introduced his famous dilemma [16]. In the early stage of development, it is still possible to influence the technology itself, but it is difficult to anticipate how the technology will impact society. Whereas, once the technology has been developed and implemented, it is difficult to influence and undo its impact. Ethical analysis can be conducted in several phases of technological development. Reijers and colleagues categorize three phases for such ethical analysis: (1) ex ante approaches, dealing with non-existing or emerging technologies, (2) intra approaches, dealing with technology design, and (3) ex post approaches, dealing with the ethical analysis of existing technologies [17]. There is increasingly a need to provide real time ethical guidance to emerging technologies. Ethics parallel research focuses on the early phases of technological development (phase 1 and 2) to provide upstream or midstream ethical analysis. In the early phases of technological development, important morally relevant decisions are made that influence the design as well as the effects, roles and accessibility of these technologies. Ethical analysis in these early phases aims to explicate and reflect upon these morally relevant decisions, in order to identify the underlying assumptions, implicit goals and possible effects of the technology. Ethical analysis can help in explicating hidden normativity with regard to foreseen users, in explicating goals and effects, and possibilities for abuse and unintended effects [18]. Ethical analysis of such upstream phases, is helpful to formulate conditions under which technological innovations are acceptable and desirable. It thereby overcomes the trap of being “too little too late” and enables the ethicist who is engaged in the innovation process to guide the innovation processes and governance in a morally sound way.

For example, in the past years a new type of biobank has emerged: organoid biobanks (see Supplemental file 1). The storage of organoids in so-called ‘living’ biobanks will serve the combined goals of future research and clinical purposes [19, 20]. This mixed-model of biobanking brings along a distinct set of ethical challenges that should be addressed proactively, before it becomes impossible to reverse a socially embedded, inert technology. Biobanks are an excellent example to illustrate the need for guidance during the development, as traditional governance methods such as informed consent and ethics committee review are ill-suiting to the open ended research of biobanks and ill-fitting to the ethical challenges that come with the particularities of organoids. After all, the creation, long-term storage, and exchange of organoids means that new value is generated out of ‘ordinary’ bodily material. Human tissues are transformed into biotechnological artefacts that can grow, expand and be stored limitlessly, some organoids are even immortal, and they have considerable scientific, clinical, and commercial value [19–21]. Consequently, these organoids are of (sometimes conflicting) interest to various parties, which may raise integrity conflicts and other ethical challenges. Among these parties are donors, patients, researchers, clinicians, industry and the wider public. Moreover, organoids may acquire distinct types of moral value, particularly if they resemble human tissues for which research use is ethically controversial and under different legal jurisdictions, such as brain organoids, gonadal organoids, and self-organizing embryo models (e.g. gastruloids) that mimic early human embryonic development [22]. The governance of such organoid biobanks should be organized in a manner that suits the goals, stakeholders and values at stake. Several projects have been set up to include stakeholders of organoid biobanks in an early phase, in order to align the design and governance of these biobanks with researchers, patient organizations, private parties (biotech) and ethicists (see also 5 participatory design) [23]. One example to achieve ongoing involvement during the biobanking activities is for example working with ‘consent for governance’, that entails an initial consent procedure that provides donors with information on governance and shifts the ethical emphasis from initial consent to ongoing governance obligations, which include protection of donor privacy, participant engagement, benefit sharing and oversight [21]. The benefit of ethical reflection in upstream or midstream phases is that the governance and design can still be influenced and therefore shaped to strike a balance between the sometimes conflicting interests between various parties, and to enable this kind of research in a trustworthy way. The success of this step also relies heavily on the reflexivity and willingness of researchers to integrate perspectives and interests of ‘outsiders’ in ‘their’ technology (see also 3: ethics from within). An example is the H2020 HIT-CF project, which amongst others aims to build a European organoid biobank for rare CF mutation, in which the ethics and governance is built-in proactively (https://www.hitcf.org/).

(3) ***Ethics from within***

Ethics parallel research is conducted from within; this means that the ethicist should be embedded and work together with researchers and experts developing new technologies. To identify the ethically relevant aspects and implications of new technologies parallel to the development, it is crucial to understand the specific technology, which mechanisms are essential to its functioning, and to stay up to date with new insights and on-going experiments. Ethics from within means that ethicists collaborate with bench-scientists, data-scientists or physicians to pro-actively study and understand emerging technologies and to move beyond the often rather speculative discourses that surrounds new and emerging technologies in the societal and academic discourse [24]. By studying biomedical innovations in the settings where they are developed, discussed and experimented with, reflexivity and awareness of normative implications can be built in the development process, so that possible effects and implications of the technology can be better anticipated and steered [25, 26] . By analysing and explicating such normative implications along the design process in close collaboration with the scientists, clinicians and developers, the ethicist becomes engaged in reshaping or redesigning the technology into more desirable products. Ethicists can also play a role in identifying desirable contexts and applications of the technology. Furthermore, such collaboration can help identifying safety-mechanisms that can be built into the technology and challenges that demand institutional oversight or a procedural solution, such as governance to simultaneously steer the use of such technologies in desirable directions, while remaining flexible to the further development of the technology along the way.

For example, for the development of biobanks for complex tissues, such as organoids, the scientific and clinical applications of such tissues need to be understood in order to assess the ethical dimensions, possible challenges and solutions. By working from within, the ethicist is able to provide realistic guidance to the researchers and simultaneously steer the societal debate away from far-fetched hypothetical scenarios. As indicated above, the applications and manifestations of organoids are multiple and the development of new types of organoids and possible applications goes rapidly. By working together with researchers and other experts, the ethicist is not only up-to-date about new insights and applications of these type of complex tissues, but the ethicist can also help in shaping the ways organoids are stored and used [20]. These aspects are important to formulate conditions and models, among which the above mentioned consent for governance models that enables meaningful consent procedures, a policy for recontacting donors, and terms for the use and ownership of such materials.

(4) ***Empirical research***

Technologies and innovations in biomedicine commonly focus on enabling new types of research, improving care or preventing illnesses. Once these technologies are developed, they will interact with users of such technologies, such as patients, healthy citizens or health care professionals. Technologies and innovations in biomedicine differ from other technologies in the sense that they influence our bodies, health (care) and wellbeing, and also our perceptions of health and disease. This specific context, demands extra scrutiny and alignment with end-users perspectives, given that the effects of such technologies will affect pertinent and private aspects of their lives. The perspectives of patients and other societal stakeholders are helpful for understanding how such technologies may be used or which effects it may have in practice, given that end-users have a particular type of knowledge [27, 28]. Ethical guidance to steer the design of new health technologies is enhanced by including user’s perspectives [28–30]. Empirical ethics research - such as interviews, focus groups and surveys - constitutes an appropriate way to explore user’s moral beliefs, intuitions and reasoning. Ethical theory and clinical or ‘bed-side reality’ are complementary and are both important sources of knowledge for providing ethical orientation for such applied questions [31, 32]. Foreseen users of the technology have knowledge about their every-day life that is helpful for identifying potential effects and implications of the implementation of the new technology and how the technology can be aligned with the user’s needs, such as the workflow of a clinician or the routine of a patient. Similarly, during the first use of new technologies, empirical research can help in analysing how the technologies change (users’) values and realities (see also 5: participatory design), and whether the intended use matches the actual use of the technology.

For example, interviews with organoid-donors with Cystic Fibrosis have provided useful insights into how donors perceive their relation to the organoid. Many donors express an ambiguous relation towards organoids [33]. They see organoids as both closely and distantly related to them, influenced by the material nature of organoids, the intended type of application and the donor’s motives for donating [33]. These insights help in formulating conditions under which patients are willing to donate their material and to articulate appropriate governance for the storage and use of organoids in biobanks. These insights also inform more abstract debates about the moral status of complex tissues and ownership about bodily tissue and can be used to design consent forms for organoid biobanks.

(5) ***Participatory design***

Technologies in biomedicine (should) serve societal goals such as prevention or treatment of illnesses and the relief of pain and suffering. Because of this integration of societal goals in technologies, the development of these technologies is no longer perceived to be external enterprise, but intrinsically embedded in society [12]. This means that societal actors have a stake at scientific developments. Involving members of society in participatory design can thereby contribute to the legitimization of the development of new technologies, which is a different legitimization than the knowledge-based justification for empirical research as outlined under ingredient 4. Participatory design has close ties with the notion of co-production that originates from STS and refers to the simultaneous processes through which modern societies form their epistemic and normative understandings of the world [34]. In ethics parallel research, participatory design means that patients or publics are not only asked for their opinion or understanding, but they are involved in several or all steps of the research: they have influence on what the research is about, how it will be developed, who will be recruited and how the results can be translated into practice. Ethics parallel research draws on participatory research to focus on the reasons, goals and motivations that drive technological innovation and to evaluate whether the driving forces align with societal needs (see also section 6: societal impacts). The level of participation can vary, based on the questions, phase of development and feasibility and should be designed to navigate between inclusiveness and demandingness of participatory practices [35].

An example for the value of participatory design can be found in the development of AI (See also Supplemental file 1). It is increasingly recognized that AI has the potential to radically change multiple aspects of our society: How we do business, how we teach and learn, our work environment and tasks, our interactions with other humans, and also the healthcare we give and receive. The ethical implications of the application of AI in medicine go much further than mere technical questions about safety and effectiveness., Medical AI may change the practice of physicians and thereby influence the patient-physician relationship, it may not be understandable how the underlying algorithms come to certain recommendations and it has been widely recognized that if we are to develop AI, it should be contributing to the common good [36]. Given the expected extent and wide-spread influence of AI on basically all aspects of society, publics should be included in decisions about the development, design and use of AI. Especially the participation of publics for the application of AI within medicine, is precarious, given the central role that health and wellbeing plays in attaining other societal goals. Participatory design can help to align the goals of these technologies, with what constitutes the common good and helps to identify goals and motivations that should drive the further development of medical AI, see for example the RAIDIO project (https://www.nwo.nl/onderzoek-en-resultaten/onderzoeksprojecten/i/58/35158.html).

(6) ***Societal impacts: hard and soft impacts***

The midstream of technological development is a crucial step, because it moves the technology into society. This means that first tests are performed in practice, for example by a pilot study, first-in-man study or a first field trial. It is also an exciting phase for the ethicist, because the analysis can shift focus from *intended use* to *actual use*. Traditional analysis of such early phases focus strongly on safety, harm and (cost)-effectiveness, which are in the literature sometimes referred to as ‘hard-impacts’ [37]. Hard impacts, such as costs and risks, are generally relatively easily quantifiable. For example, an appropriate balance of benefits over risks is one of the cornerstone requirements of clinical research ethics and can be classified as a typical hard impact. While hard impacts are important, focusing solely on these aspects of technologies will not provide a comprehensive picture of the diverse societal implications of technologies. Ethics parallel research, therefore, also focuses on the unquantifiable effects and more complex evaluations of new technologies. These effects, sometimes referred to as ‘soft impacts’, focus on the influence of new technologies on our values such as autonomy and human flourishing, experiences, identity, relations and perceptions [37–39]. The analysis of hard and soft impacts helps to provide orientation to a comprehensive understanding of the most desirable ways of dealing with and interacting with new technologies. The analysis of hard and soft impacts can be informed by empirical research, but can also be a mere analytical exercise that draws on examples and bioethical theories.

To give an example: one of the pressing questions in the ethical and societal debate on the implementation of AI in medicine, is how humans and AI should cooperate. While AI is able to deal with an overwhelming amount, production speed and multidimensionality of data, that well exceeds the limits of understanding of the human brain [40], the role of AI in clinical judgement and in clinical decision-making is far from clear. The role AI will take will, however, influences its societal acceptability. Similarly, the ways in which AI can integrate diverging patient values and respect individual patient’s autonomy have been raised as important conditions for the implementation of medical AI [41]. In order to comprehensively evaluate the desirability of implementing medical AI, we should focus on both its soft and hard impacts.

## Conclusion

Ethics parallel research brings together elements from traditional bioethics, learned from the ‘empirical turn in bioethics’ [27] and from approaches of STS and philosophy of technology including Responsible Research and Innovation (RRI) [42]. It is, as we described, characterized by six related and even overlapping ingredients that can be conducted in somewhat overlap next to each other: (1) it concerns disentangling of wicked problems, (2) it is upstream or midstream (3) it is “ethics from within”, (4) it includes empirical research, (5) draws on public participation and (6) it focuses on societal impacts, including hard and soft impacts. While these six ingredients are increasingly adapted by bioethicists and are not new by and in themselves, similarly like in cooking, the sum of these six ingredients is more than their parts. It should be noted that one does not need all ingredients all the time to still have a lovely meal – indeed ethical analysis of new biotechnologies can be conducted without combining all six ingredients. Together these six ingredients, constitute ethics parallel research, even if they can vary in terms of intensity depending on the particular technology. The combination of these six ingredients allows to fulfil two aims: guiding the development process of technologies in biomedicine and providing input for the normative evaluation of such technologies. With regard to the former: The ethicist aims to guide technology that is not fully developed yet, this means that a typical source for ethical evaluation, namely morally relevant experiences with a practice or technology, is not available (yet). To guide the development, including the design, of technology parallel or pro-actively, the ethicist needs to anticipate possible effects, by drawing on multiple sources, such as information from the developers, publics and end-users (which are often clinicians and patients). Simultaneously, ethics parallel research interacts with societal stakeholders as the development of new technologies and should be responsive and aligned with societal values.

By following the ethics parallel approach, the ethicist is able to identify and evaluate ethical questions already in the early phases of technological development and to constructively guide the development of new technologies, by bringing reflexivity in the design process and information and structure to the societal debate. This expands, rather than replaces, the types of evaluation and roles that bioethicists have in more traditional approaches, where ethicists evaluate developed technologies, evaluate whether technologies are aligned with existing guidelines or speculate and reflect on questions whether the development of a non-existing technology would be desirable. Moreover, a distinctive aspect of ethics parallel research is a constructive, rather than a pure critical outlook: it aims to develop and contribute to best-practices rather than outlining worst practices. Importantly, ethics parallel research does not aim to replace more traditional approaches for developing normative theories and principles, but it results in both empirical and more theoretical insights that can provide the input for normative analysis and the formulation of more abstract normative recommendations. The formulation of such normative recommendations remains certainly also an important task for bioethicists, and would require an additional approach. The insights obtained by the six ingredients can for example be used as input in the widely adopted wide reflective equilibrium [30, 43].

Ethics parallel research has, like any approach, also drawbacks and pitfalls. First, it is an approach to ‘think with’, rather than (only) ‘think about’ practice. This means that the ethicist is ‘embedded’ in technological and scientific practice and must take care to avoid ‘going native’. Embedded ethicists will need to navigate between staying embedded and finding a critical constructive voice. As with other potential conflicts of interest, ethicists should be transparent about the nature of the collaboration and potential (financial) support. In addition, it may be helpful to also involve an external ethicist to the project when proposing specific guidelines [44]. A related point is that for the outside world the ethics parallel researcher may be perceived as a window dresser for technological developments, because in contrast to the more traditional image of an ethicist who outlines what is wrong with a certain technology, ethics parallel research works closely together with developers of technology and aims to enable and improve technological development. One has to stay alert to stay distant from so-called “ethics washing”.

Second, morality does not only influence technology, but also vice versa. As the technology influences societal values during the technological process, the values identified more upstream in the development may differ from the values with which the technology is evaluated more down-stream of the development. Due to so-called techno-moral change, the moral yardstick to assess the desirability of a certain technology may change along the development of the technological innovation. The goal of ethics parallel research is not to formulate conditions for technological innovation that are carved in stone, but to remain flexible and reflective throughout the development process so that ethical guidelines remain fit for purpose, rather than a dogma. The benefits of ethical guidance during the development process should, however, not be underestimated: mitigating negative effects of technologies, enhancing positive impact and sensitizing researchers and developers to the ethical and societal impact of their research, are in themselves desirable aims within applied ethics.

Third, the development of new technologies is unpredictable, some technologies develop faster than foreseen, others fail unexpectedly, while still others may have unforeseen and unintended impacts. These issues relate to the anticipation side of the Collingridge dilemma: in the early stages of development, meaningful guidance is very difficult. The aim of ethics parallel research to provide guidance in the early phases of technological development, therefore brings forward the challenge of finding the right time to jump on and off board. The timing will much depend on the specific technology and the way in which it will develop. Ethicists will have to learn to recognize the right time and widen their skillset to make sense of the early phases of technological innovations. In addition, they will have to adapt ways of exploring expectations, needs and anticipation of patients and other societal stakeholders with regard to new (bio) technologies, rather than focusing solely on their experiences. Only then meaningful orientation and anticipatory governance can be formulated to meaningfully guide development of new (bio)technologies.

In this paper we have drawn on three examples, organoids, gene editing and AI to illustrate the types of issues ethics parallel research is suited for. These examples vary greatly in terms of their development, societal implications and wickedness. We believe that ethics parallel research is a valuable approach for guiding the development of these technologies, and virtually any other innovation within biomedicine.

To conclude, ethics parallel research is an approach that is well-suited to the ethical evaluation of biomedical innovations. It means that ethicists identify and evaluate the ethical issues of a novel biomedical technology parallel or even proactively as the field develops. By bringing together ingredients that orginate from traditional bioethics, empirical bioethics, STS and philosophy of technology, this approach can ethically guide the increasing permeation of technologies and innovations in the field of biomedicine. It widens the roles and judgements from the ethicist to a more anticipatory and constructively guiding role. The benefits of this approach are to provide guidance along the process of biomedical innovation, to sensitize (bio) medical scientists for the ethical aspects of their work, and to mitigate negative effects.

## Supplementary information

**Additional file 1: Supplemental File 1.** Description of the technologies used to illustrate the approach of ethics parallel research

## Data Availability

Not Applicable.

## Abbreviations

AI: Artifical intelligence
CRISPR: Clustered regularly interspaced palindromic repeats
