# Supplementary material for: Ethics parallel research: an approach for (early) ethical guidance of biomedical innovation
Source: BMC Med Ethics. 2020 Sep 1;21:81. doi: 10.1186/s12910-020-00524-z (PMC7461257; doi:10.1186/s12910-020-00524-z)
Supplement: Supplementary file 1 — Additional file 1: Supplemental File 1. Description of the technologies used to illustrate the approach of ethics parallel research [file 12910_2020_524_MOESM1_ESM.docx]

**Supplemental File 1:** Description of the technologies used to illustrate the approach of ethics parallel research

Gene editing: Gene, or genome, editing is a type of genetic engineering in which DNA is inserted, deleted, modified or replaced in the genome of a living organism. Unlike early genetic engineering techniques that randomly insert genetic material into a host genome, genome editing targets the insertions to site specific locations. Genome editing was revolutionized by the emergence of Clustered Regularly Interspaced Palindromic Repeats (CRISPR) and the CRISPR-associated protein 9 (Cas9). Aside from possibilities to alter the germline genome for inheritable diseases, new applications of these genome editing technologies have emerged, such as synthetic gene drives, which allow the rapid and super-Mendelian spread of gene alterations within a population or even a species.

Organoids: Organoids are three-dimensional self-organized tissue cultures that are derived from stem cells. Organoids can be studied as models for how organs develop and grow, and can be used as a model to test medication. Several applications are developed: Organoids that resemble the gut are for example used in the context of precision-medicine for Cystic Fybrosis and stored in biobanks, the development of brain organoids can be used to study the biological aspects of psychiatric conditions, liver organoids are developed for transplantation, whereas gastruloids, that resemble early stage embryos, could provide insights into early embryonic development.

AI: Artificial intelligence is the field of research that focuses on the development and understanding of intelligent computational processes. AI and other types of machine learning, for example the clever linkage of algorithms, can help computers to solve problems or recognize patterns in large sums of data, as good or even better than humans can. AI application in health care are various, but most prominently AI is now being implemented in diagnostic fields that are image-based, such as radiology and pathology.
